# Dicer Deletion in Astrocytes Inhibits Oligodendroglial Differentiation and Myelination

**DOI:** 10.1007/s12264-021-00705-9

**Published:** 2021-06-09

**Authors:** Kun Liu, Bin Yu, Jing-Fei Chen, Rui-Xue Li, Lin Chen, Shu-Yu Ren, Fei Wang, Feng Mei, Lan Xiao

**Affiliations:** 1 Department of Histology and Embryology, Chongqing Key Laboratory of Neurobiology, Brain and Intelligence Research Key Laboratory of Chongqing Education Commission, Third Military Medical University (Army Medical University), Chongqing, 400038 China; 2 Department of Neurosurgery, 2nd affiliated Hospital, Third Military Medical University (Army Medical University), Chongqing, 400038 China

**Keywords:** White matter, Myelination, MicroRNA, GFAP, MT/mG, Microglia, Oligodendrocyte, Demyelination, Remyelination

## Abstract

Increasing evidence has shown that astrocytes are implicated in regulating oligodendrocyte myelination, but the underlying mechanisms remain largely unknown. To understand whether microRNAs in astrocytes function in regulating oligodendroglial differentiation and myelination in the developing and adult CNS, we generated inducible astrocyte-specific Dicer conditional knockout mice (hGFAP-CreERT; Dicer fl/fl). By using a reporter mouse line (mT/mG), we confirmed that hGFAP-CreERT drives an efficient and astrocyte-specific recombination in the developing CNS, upon tamoxifen treatment from postnatal day 3 (P3) to P7. The Dicer deletion in astrocytes resulted in inhibited oligodendroglial differentiation and myelination in the developing CNS of Dicer cKO mice at P10 and P14, and did not alter the densities of neurons or axons, indicating that Dicer in astrocytes is required for oligodendrocyte myelination. Consequently, the Dicer deletion in astrocytes at P3 resulted in impaired spatial memory and motor coordination at the age of 9 weeks. To understand whether Dicer in astrocytes is also required for remyelination, we induced Dicer deletion in 3-month-old mice and then injected lysolecithin into the corpus callosum to induce demyelination. The Dicer deletion in astrocytes blocked remyelination in the corpus callosum 14 days after induced demyelination. Together, our results indicate that Dicer in astrocytes is required for oligodendroglia myelination in both the developing and adult CNS.

## Introduction

Astrocytes are a type of glial cell that are widely distributed in the central nervous system (CNS) [1]. It has been well established that astrocytes play important roles in CNS development and injuries, such as providing nutrients to neurons [2–4] and responding to injury and inflammation [5, 6]. Oligodendrocytes (OLs) wrap axons and form myelin sheaths that ensure fast and efficient transmission of action potentials in the CNS [7, 8]. The OL differentiation and myelination are initiated by axon-OL recognition, and this process is also guided by interacting with other cell types [9, 10]. Indeed, astrocytes are closely associated with OLs by communicating through gap junctions and secretory factors [11, 12]. *In vitro* evidence has shown that astrocytes can influence the differentiation, proliferation and migration of OL precursor cells (OPCs) [13]. For example, astrocytes secret growth factors that can promote OPC proliferation and survival in cultures [14]. Elimination of astrocyte in the developing CNS causes OL death and disrupts myelination in the developing CNS, indicating that astrocytes are functionally required for OL survival [15]. However, *in vivo* mechanisms of astrocytes that regulate OL myelination remain largely unknown during CNS development and injuries.

Dicer is a key enzyme that is responsible for generating functional noncoding micro-RNAs (miRNAs), that bind to and degrade target message RNAs. The functional importance of Dicer in OLs and neurons has been extensively investigated. Conditional deletion of Dicer driven by mGFAP-Cre or hGFAP-Cre in the developing CNS results in premature death of the knockout mice and disrupted synaptogenesis in developing brains [16, 17]. It is noticeable that the mGFAP-Cre and hGFAP-Cre lines induce non-selective recombination in neurons, OLs, and astrocytes [18], due to the GFAP expression in embryonic neural stem cells [19]. Since myelination occurs in the CNS postnatally and continues throughout life in mice, it is still unknown whether the expression of Dicer in astrocytes is required for OL differentiation and myelination.

In this study, we used the hGFAP-CreERT mouse line to induce recombination in astrocytes from postnatal days 3–7. The recombination occurred efficiently and specifically in astrocytes. By crossing the hGFAP-CreERT mouse to the Dicer floxed (Dicer fl/fl) mouse, the induction of Dicer deletion in astrocytes resulted in delayed OL differentiation and myelination, and subsequently caused spatial memory and motor coordination deficits at the age of 9 weeks. To understand whether Dicer is required for remyelinaiton, we induced Dicer ablation in astrocyte at the age of 3 months and then demyelination by injecting lysolecithin into the corpus callosum. Consistently, the remyelination was also inhibited in the corpus callosum of Dicer cKO mice compared to wild-type controls 14 days after lesion. Together, our results indicate that Dicer in astrocytes is required for OL differentiation and myelination and astrocyte may play an important role in regulating OL myelination.

## Materials and Methods

### Animals

Mice used in this study were handled according to the protocols approved by the Laboratory Animal Welfare and Ethics Committee of the Army Medical University (Third Military Medical University). The hGFAP-CreERT mice were a generous gift from Dr. Christian Giaume (MGI:4418665) and were described previously [20]. To generate astrocyte-specific Dicer cKO mice, the hGFAP-CreERT2 mice were bred with the Dicer fl/fl line (The Jackson Laboratory, Catalog # 006366) to obtain hGFAP-CreERT; Dicer fl/fl mice. The reporter gene mice mT/mG were purchased from The Jackson Laboratory (Catalog # 007676) and crossed with the hGFAP-CreERT line. Genomic DNA was extracted from mouse tails and genotypes of all mice were analyzed by PCR with the according primers. Dicer fl/fl mice or hGFAP-CreERT; mT/mG littermates were used as controls.

### Administration of Tamoxifen

To induce Cre recombination in mice, Tamoxifen (Sigma-Aldrich, St. Louis, MO) was dissolved in sunflower oil to a final concentration of 10 mg/mL. Newborn pups and adult mice were administered tamoxifen at 50 mg/kg per day for five consecutive days by oral gavage.

### Immunofluorescence Staining

Immunofluorescence staining was carried out as described previously [21]. Briefly, floating tissue sections (20 μm) were prepared from 4% paraformaldehyde perfused brains and were blocked with 5% bovine serum albumin (BSA) containing 0.4% Triton-X 100 for 1 h at room temperature. Then tissues were incubated with primary antibodies to rabbit anti-NG2 (Millipore,1:200), rabbit anti-BLBP (Abcam, 1:1000), rabbit anti-Olig2 (Millipore, 1:200), rabbit anti-NeuN (Abcam, 1:500), rabbit anti-NF200 (Sigma-Aldrich 1:1000), rat anti-MBP (Millipore 1:200), mouse anti-CC1 (Calbiochem 1:200), rabbit anti-Iba1 (Wako 1:500), or goat anti-GFAP (Abcam, 1:500) overnight at 4°C. After thorough washing, appropriate secondary antibody (Alexa Fluor 647 donkey anti-mouse IgG, Alexa Fluor 647 donkey anti-rabbit IgG, Alexa Fluor 647 donkey anti-goat IgG, or Alexa Fluor 647 goat anti-rat IgG; Molecular Probes, Invitrogen) was applied at 1:1000. Nuclei were counterstained with 4’, 6-diamino-2-phenylindole (DAPI, Invitrogen).

### Morris Water Maze (MWM)

The water maze is a behavioral test designed to assess the spatial learning skill [22]. The test apparatus consists of a circular water tank filled with opaque water and a hidden platform submerged a few centimeters under the water surface in one quadrant of the tank. In the training trials, mice were place into the water at different quadrants to find the hidden platform at a fixed position. All mice were tested four times a day for 3–5 consecutive days. In the test trials, the platform was removed and distance moved in platform quadrant, time spent in the platform quadrant, or times crossing the platform area was measured to assess long-term spatial memory. Investigators were blinded for the genotype information and mice were handled gently to avoid stress in the experiment.

### Beam Walking Test

This test was applied to measure hind limb motor coordination. A 0.5 cm wide beam was placed 50 cm over the floor in a dark room, the starting side was lightened by a lamp and the other side was in a box (non-transparent, 20 cm × 10 cm × 20 cm). Padding material from cages was placed on the bottom of the box to attract the mice to walk through. Each mouse was put on the beam in a sequence of 30-cm, 50-cm, and 70-cm from the end during the training day. And this kind of training was repeated two times per day for 3 consecutive days before testing. On the testing day, videos were recorded from both sides of the beam. The frequency of hind limb slippage was measured as the index of the beam walking test when the mice walked along the 90-cm long beam [23].

### Open Field Test

The open field test was used to measure motor capacity by using an open field activity system (Biowill, Shanghai, China). Mice were placed in the center of the uncovered box (50 cm × 50 cm × 50 cm), and their movements were recorded for 10 min as they moved around and explored the environment. After the experiment was completed, the distance traveled was measured by a computer tracking programs.

### Image Acquisition and Quantification

Fluorescent images were obtained with an Axio Imager M2 fluorescence microscope (Zeiss, Oberkochen, Germany) and a confocal laser-scanning microscope (Olympus, FV 3000, Shinjuku, Tokyo) at appropriate excitation wavelengths. For statistical analysis, at least three representative images (20×) from comparable locations were acquired for each sample. Quantification was analyzed using Image-Pro Plus 6.0 software (Media Cybernetics, Silver Spring, MD, USA).

### Lysolecithin Demyelination Model

Three-month-old mice (Dicer cKO or control) were anesthetized with isoflurane, and the skull was fixed horizontally during injection. A steel needle (30 GA, Hamilton) was attached to a 10 μL Hamilton syringe by a microtube segment, and installed on a stereotaxic micromanipulator (RWD). 1.5 μL of 1% lysolecithin (Sigma-Aldrich) solution was injected unilaterally into the corpus callosum (1.00 mm posterior to bregma, 1.04 mm lateral to bregma,1.58 mm deep) to induce focal demyelination. The needle was kept in place for 5 min to reduce reflux along the needle track. 2 weeks later, mice were sacrificed and their demyelination and remyelination were assessed [24].

### Electron Microscopy

Electron microscopy (EM) was performed as previously described [25]. Briefly, animals were anesthetized with 1% pentobarbital and perfused with 1.25% glutaraldehyde/2% paraformaldehyde in 0.1 mol/L PB, pH 7.4. Tissues were postfixed with 1% OsO_4_, incubated with uranyl acetate, dehydrated through a graded acetone series and embedded in epoxy resin 618. Ultrathin sections (60 nm) were cut by an ultramicrotome (LKB-V, LKB Produkter AB, Bromma) and observed under a transmission electron microscope (TECNAI10, Philips). High-resolution images were taken with an AMT XR-60 CCD Digital Camera System and analyzed with Image-Pro Plus 6 (Media Cybernetics, Silver Spring, MD, USA). The lateral horn white matter of the spinal cord at cervical levels 2–5 was collected for semi-thin sections. The g-ratios of the myelinated fibers were calculated as the ratio of the diameter of the axon to the diameter of the myelin sheath wrapping the axon.

### Statistical Analysis

The statistical significance of differences between groups was determined using GraphPad Prism 7 software (La Jolla, CA [https://www.graphpad.com/]). All values are expressed as the mean ± SEM. Data from the acquisition phase in the Morris water maze were analyzed by separate repeated measures two-way ANOVA. For other data, statistical analysis was performed using the two-tailed unpaired *t*-test. Each experiment included at least three samples. *P*-values less than 0.05 were considered statistically significant. Significance was reported as *P < 0.05, **P < 0.01, or ***P < 0.001.

## Results

### Astrocyte-Specific Recombination Driven by hGFAP-CreERT

**Fig. 1.**
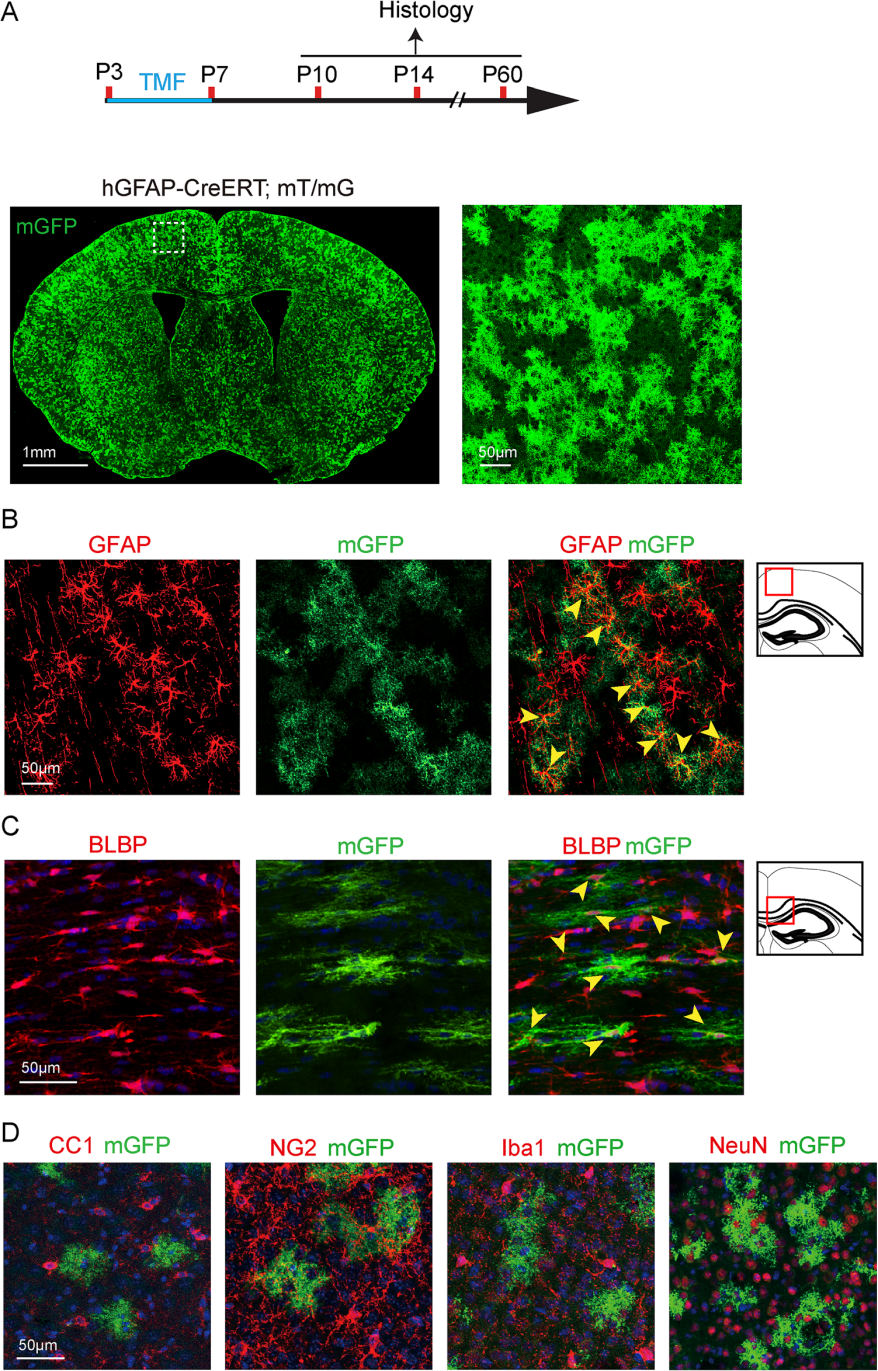
Recombination in hGFAP-CreERT; mT/mG brains. **A** Schematic diagram displaying the time course of tamoxifen (TMF) treatment and histology. Representative image of mGFP-positive cells in the brain at P14 (left), and magnified mGFP-positive cells in the cortex (right image corresponding to the dotted area in the left panel); **B**, **C** Counter-immunostaining for GFAP (yellow arrowheads indicating astrocytes in **B**), BLBP (yellow arrowheads indicating astrocytes in **C**); **D** CC1 (mature oligodendrocytes), NG2 (OPCs), Iba1 (Microglia), and NeuN (Neurons) positive cells in hGFAP-CreERT; mT/mG brains. Scale bar, 1 mm (Left panel of **A**) and 50 µm (Right panel of **A**, **B**, **C**, and **D**).

To understand the role of astrocytes during CNS development, we used the hGFAP-CreERT mouse line to induce recombination in the developing CNS (Fig. 1). To examine the recombination specificity and efficiency driven by hGFAP-CreERT, we crossed this line to the mT(omato)/mG(FP) reporter line and mice were treated with tamoxifen at postnatal day 3 (P3) and sacrificed at P14 (Fig. 1A). The membrane-bound GFP (mGFP) expression was initiated upon recombination, and the mGFP positive cells were widely distributed throughout the brain (Fig. 1A). To verify if the hGFAP-CreERT line can induce astrocyte-specific recombination in the postnatal CNS, we carried out immunostaining for GFAP, brain lipid-binding protein (BLBP, astrocytes), NeuN (neurons), CC1 (OLs), Iba1 (microglia), and NG2 (OPCs) on the hGFAP-CreERT; mT/mG brain sections (Fig. 1B–D). Our results showed that mGFP-positive cells were exclusively co-localized with astroglial markers, GFAP or BLBP, and did not overlap with CC1, Iba1, NeuN, or NG2 expression (Fig. 1B, C). It was noticeable that approximately 85% of GFAP- and 60% of BLBP-positive cells expressed mGFP in the cortex and corpus callosum respectively, indicating that the hGFAP-CreERT line was reliable in inducing astrocyte-specific recombination in the gray and white matter of the developing CNS (Fig. 1).

### Dicer Deletion in Astrocytes Inhibits Oligodendrocyte Myelination

**Fig. 2.**
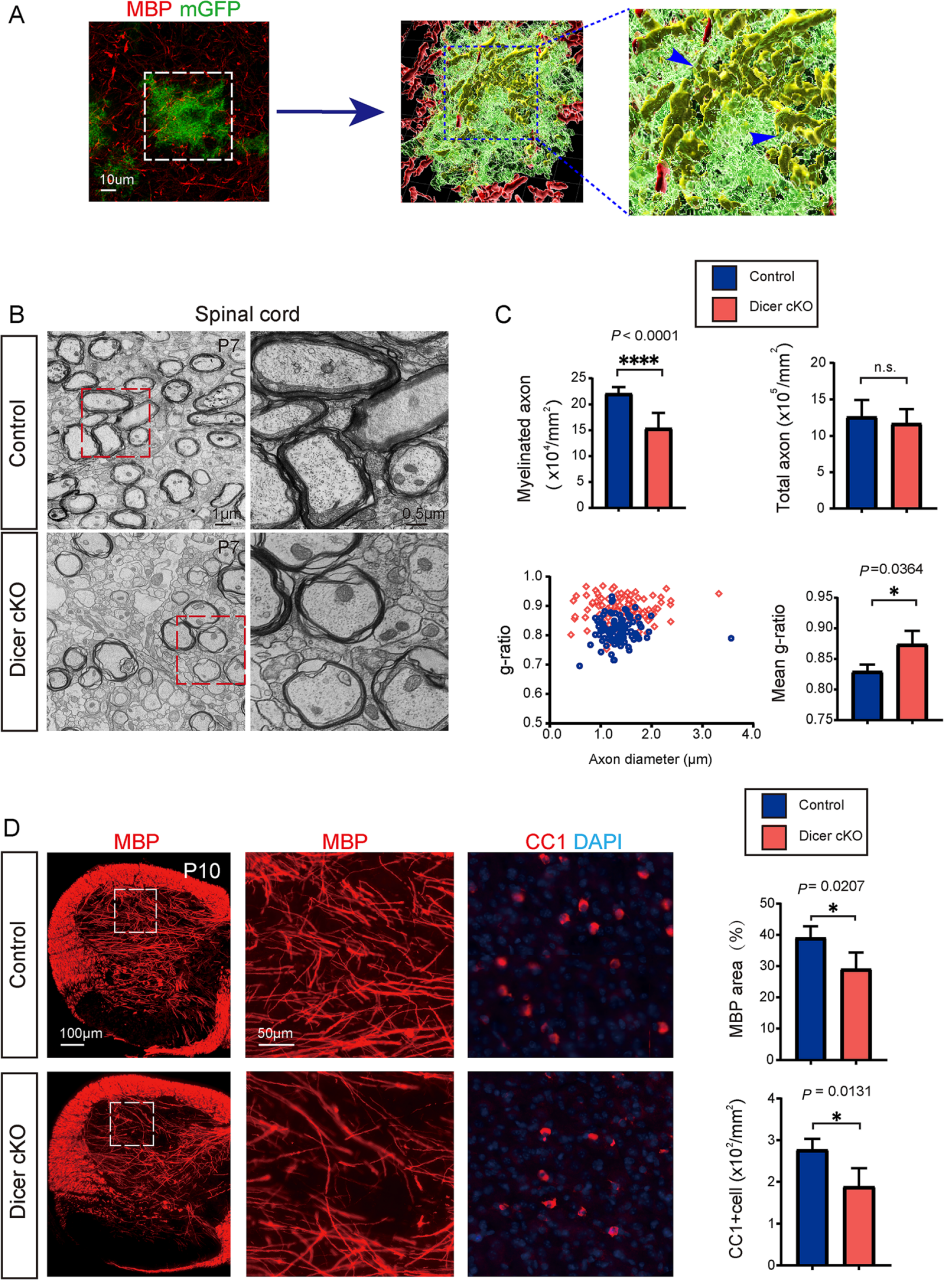
Dicer deletion in astrocytes inhibits myelination in the developing spinal cord. **A** 3D reconstruction of mGFP-positive astrocytes, MBP-positive myelin sheaths, and arrows indicating the myelin sheaths engulfed by an mGFP-positive astrocyte; **B** Representative TEM images showing spinal cord white matter in the Dicer-cKO mice and littermates (left panels) and magnified images (right panels). Scale bars, 1 µm (left panels) and 0.5 µm (right panels). **C** Quantification of myelinated axons and total axons, myelin sheath thickness, and scatterplot of g-ratios of individual axons as a function of axonal diameter. **D** Representative images and quantification of MBP expression and CC1-positive cells in the spinal cords of Dicer-cKO and control mice. Scale bars, 100 μm (left panels) and 50 µm (middle and right panels). Error bars represent mean ± SEM. *P < 0.05 or ****P < 0.0001, Student’s *t*-test, Dicer-cKO *vs* control, *n* = 4 for all experiments.

**Fig. 3.**
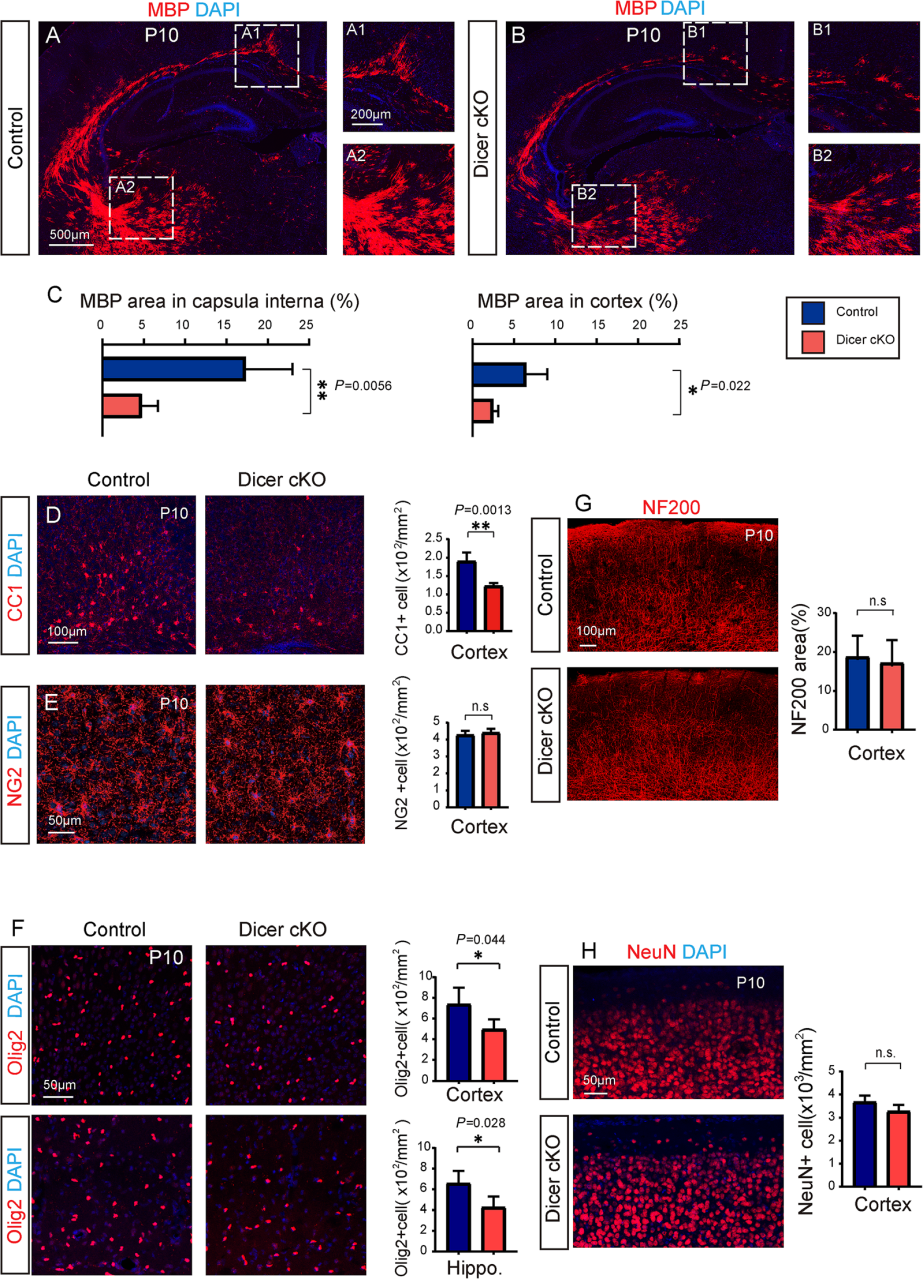
Dicer deletion in astrocytes inhibits myelination in the brain. **A, B** Representative images showing MBP-positive areas in the brains of Dicer-cKO (**A**) mice and littermates (**B**) at P10 and magnified images showing MBP expression in the cortex (**A1, B1**) and capsula interna (**A2, B2**). Scale bars, 500 µm (**A**, **B**) and 200 µm (**A1, A2, B1, B2**). **C** Quantification of MBP expression in the cortex and capsula interna. **D–H** Representative images and quantification of CC1-positive cells (**D**, **F**), NG2-positive cells (**E**, **F**), NF200 axons (**G**), and NeuN-positive neurons (**H**) in the cortex of Dicer-cKO mice and littermates at P10. Scale bars,100 µm (**D**, **G**) and 50 µm (**E**, **F**, and **H**). Error bars represent the mean ± SEM. **P < 0.01, Student’s t-test, Dicer-cKO *vs* control, *n* = 3–4 for all experiments.

**Fig. 4.**
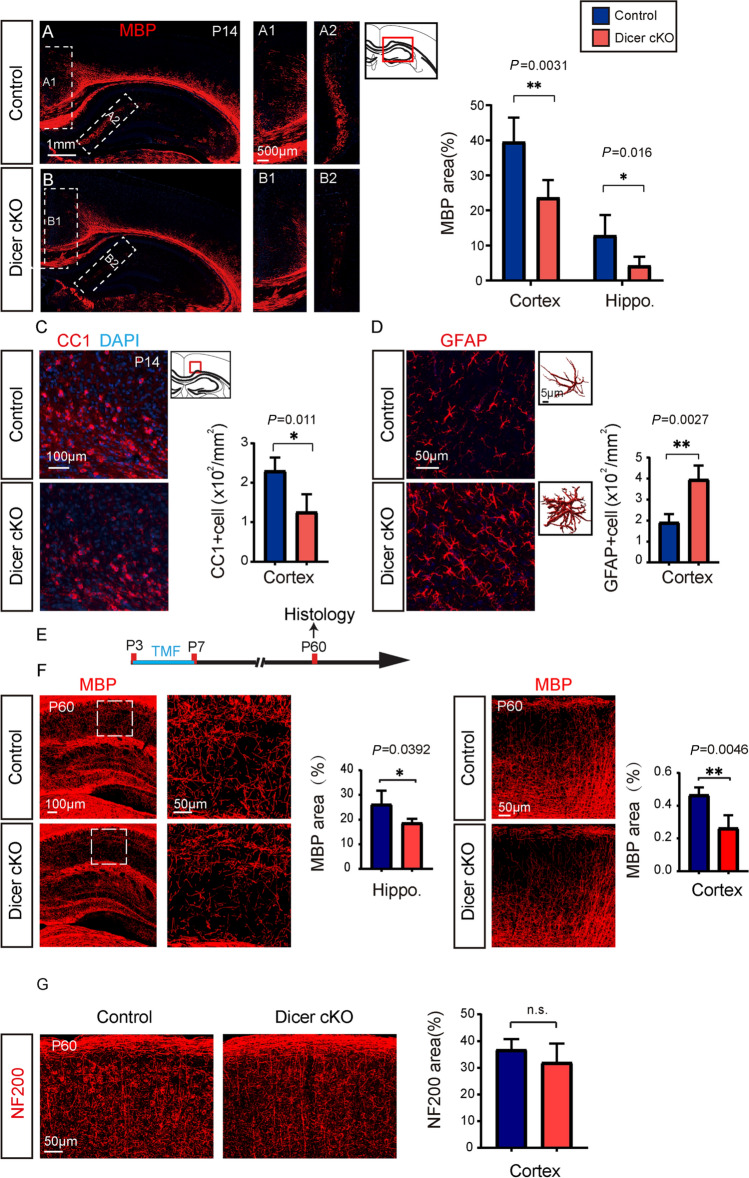
Persistent hypomyelination in the Dicer-cKO brain. **A, B** Representative images showing MBP-positive areas in the brains of Dicer-cKO (**A**) mice and littermates (**B**) at P14 and magnified images showing MBP expression in the corpus callosum (**A1, B1**) and hippocampus (**A2, B2**). Scale bars, 1 mm (**A, B**) and 500 µm (**A1, A2, B1, B2**). **C, D** Representative images and quantification of CC1- (**C**) and GFAP-(**D**) positive cells in the cortex of Dicer-cKO mice and littermates at P14. Scale bars, 100 µm (**C**), 50 µm (**D**). **E** Schematic diagram of the time course of tamoxifen (TMF) treatment and histology; **F** Representative images showing MBP-positive areas in the brains of Dicer-cKO mice and littermates at P60 and magnified images showing MBP expression in the hippocampus and cortex. Scale bars, 100 µm (left panel) and 50 µm (middle and right panels). **G** Images showing NF200-positive areas in the brains of Dicer-cKO mice and littermates. Error bars represent the mean ± SEM. **P < 0.01, Student’s *t*-test, Dicer-cKO *vs* control, *n* = 4–5 for all experiments.

Then we decided to assess whether Dicer deletion in astrocytes can change myelination in the developing CNS, since the mGFP-positive astrocytes seems to be closely associated with MBP-positive myelin sheaths in the 3D-reconstructed Z-stack images (Fig. 2A). We examined myelination by using transmission electronic microscopy (TEM), and the myelinated axon density was greatly decreased in the P7 spinal cord white matter of Dicer-cKO mice, manifested as higher *g*-ratios (Fig. 2B, C), suggesting Dicer deletion in astrocytes inhibits myelination in the developing CNS. In support of this notion, the area of MBP-positive myelin and the number of CC1-positive mature OLs were greatly decreased in the spinal cords of the P10 Dicer-cKO mice compared to wild-type controls (Fig. 2D). Consistently, MBP expression and CC1-positive cells were also significantly decreased in the P10 Dicer-cKO brains (Fig. 3A–D) without altering NG2-positive OPCs (Fig. 3E). The Olig2-positive OL density was also decreased in the Dicer-cKO brains at P10 (Fig. 3F), suggesting that Dicer deletion in astrocytes inhibits OL differentiation. The densities of NeuN-positive neurons and NF200-positive axons were not changed in the P10 Dicer-cKO cortex, indicating that Dicer deletion in astrocytes is unlikely to cause neuronal death or axonal degeneration (Fig. 3G, H). In addition, hypomyelination was still evident in the corpus callosum and hippocampus in the P14 Dicer-cKO brains revealed by MBP and CC1 immunostaining as compared to wild-type controls (Fig. 4A–C). We next examined the change of astrocytes upon Dicer deletion from P3 by immunostaining for GFAP. Our results showed increased numbers of GFAP-positive astrocytes and 3D reconstruction manifested an amplified shape of GFAP-positive cells with branched processes in the Dicer-cKO brains at P14 (Fig. 4D), suggesting that Dicer ablation in astrocytes may cause astrogliosis and activation. It is notable that the hypomyelination was persistently present at P60, as revealed by decreased MBP expression in the cortex and hippocampus of Dicer-cKO mice as compared to wild-type controls (Fig. 4E, F), while NF200 axon density was not altered (Fig. 4G). These findings indicate that Dicer expression in astrocytes is required for OL differentiation and myelination in the developing CNS.

### Dicer Deletion in Astrocytes Causes Prolonged Functional Deficits in Adulthood

**Fig. 5.**
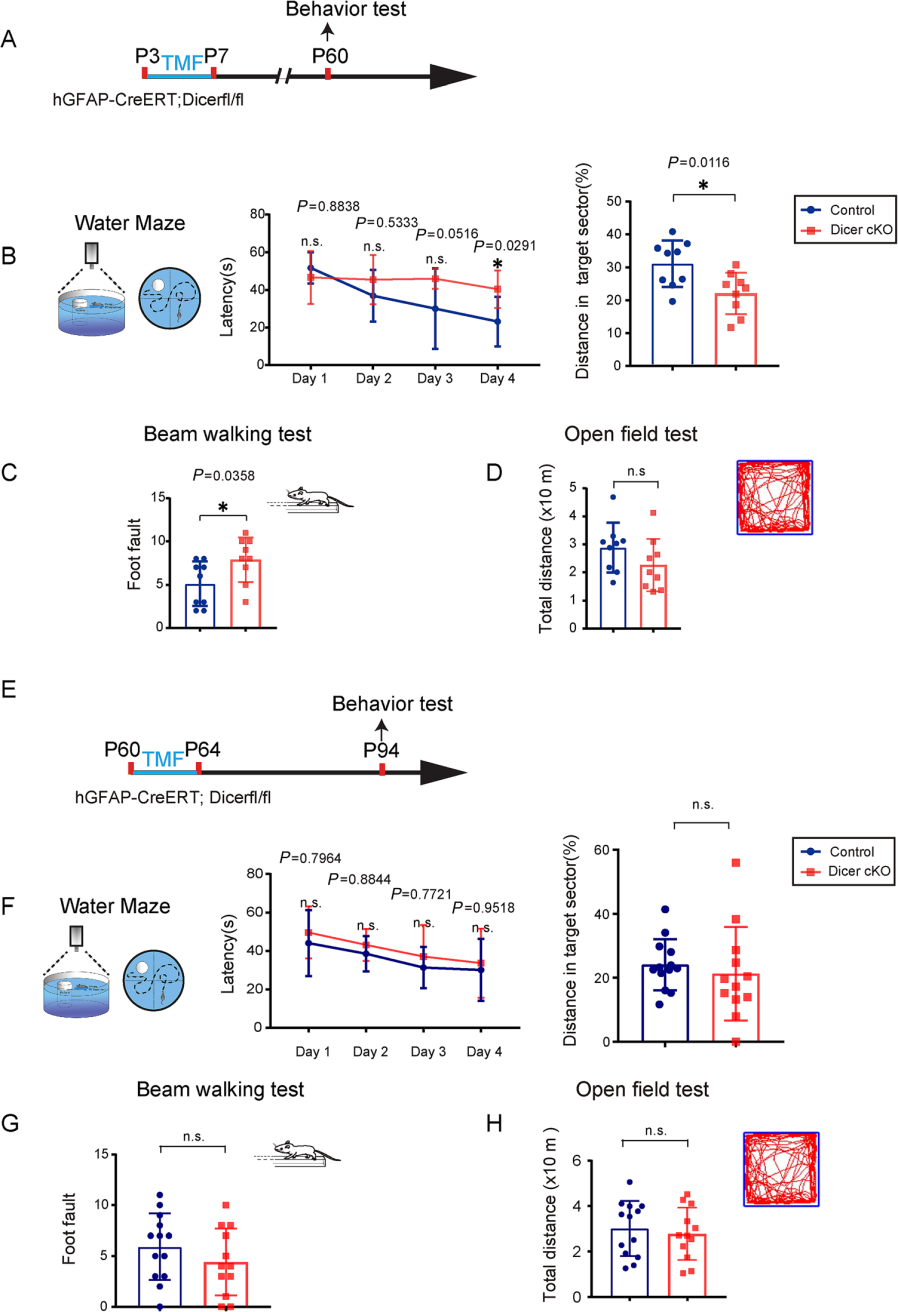
Dicer deletion in astrocytes causes memory and motor deficits. **A, E** Schematic diagram showing the time course of tamoxifen (TMF) treatment in P3 or P60 mice and behavioral tests; **B, F** The Morris water maze test reveals the latency to platform in the acquisition phase and distance spent in the target quadrant in the 60-day-old (**B**) or 94-day-old (**F**) Dicer-cKO and littermate control mice. Error bars represent the mean ± SEM. *P <0.05, unpaired *t* test and two-sided unpaired Mann-Whitney test were used for latency to platform and distance in the target sector. (**C, G**) Mean numbers of foot slips of Dicer-cKO and control mice in the beam-walking test at P60 (**C**) or P94 (**G**). Error bars represent the mean ± SEM. *P < 0.05, non-parametric Mann-Whitney test, Dicer-cKO *vs* control mice; **D, H** Total distance in the open field test at P60 (**D**) or P94 (**H**). *n* = 9 Dicer-cKO mice and *n* = 9 control mice at P60 (**B, C**), and *n* = 13 Dicer-cKO mice and *n* = 12 control mice at P94 (**D, F**); non-parametric Mann-Whitney test.

To investigate whether the Dicer deletion in astrocytes could affect neurofunction development, we examined the behavioral changes in adulthood at P60 with the induction of recombination at P3 (Fig. 5A). The Dicer-cKO mice were viable without showing visible abnormalities, and had a lifespan similar to that of littermate controls. It was interesting that the Dicer-cKO mice displayed impaired spatial memory in the Morris water maze test, with prolonged latency in the training process and lower frequency of passing the target sector (Fig. 5B). In addition, the beam-walking test displayed that motor coordination was impaired in the Dicer-cKO mice, showing a higher frequency of foot-slip than wild-type controls (Fig. 5C). To examine whether motor function was altered in the Dicer-cKO mice, the mice were subjected to the open field test, and the travel distance was not significantly different between the Dicer-cKO and littermate control mice (Fig. 5D). The results indicated that the Dicer deletion in astrocytes during CNS development caused neuronal dysfunctions in spatial memory and motor coordination in adulthood (Fig. 5). To further examine if the functional deficits were due to Dicer deletion during development or adulthood, we induced recombination in adulthood at the age of 60 days (Fig. 5E–G). One month after recombination, we challenged the Dicer-cKO and control mice with the same set of behavioral tests. The Dicer-cKO mice did not manifest significant changes in the water maze (Fig. 5F), beam-walking (Fig. 5G), or open field test (Fig. 5H), suggesting that Dicer deletion in astrocytes during adulthood may not cause instant functional deficits.

### Dicer Deletion in Astrocytes Delays Remyelination After Lysolecithin-Induced Demyelination

**Fig. 6.**
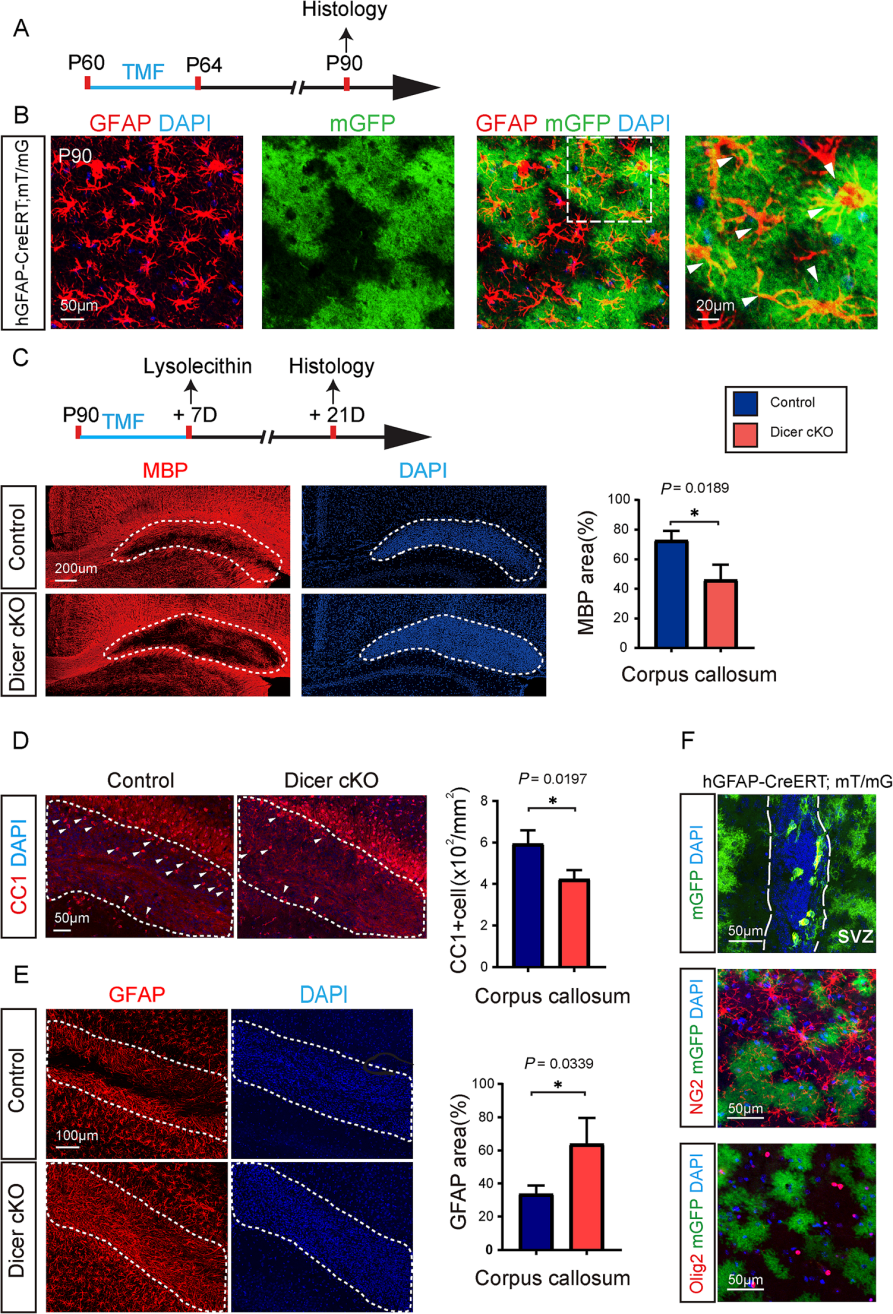
Dicer deletion in astrocytes inhibits remyelination after lysolecithin-induced demyelination. **A** Schematic diagram of the time course of tamoxifen (TMF) treatment and histology; **B** Counter-immunostaining for GFAP (white arrowheads indicating astrocytes in **B**). Scale bars, 50 μm (middle and left panels) and 20 µm (right panels); **C** Schematic diagram showing the time course of tamoxifen (TMF) treatment, lysolecithin injection, and histology. Representative images and quantification of MBP expression in the lesion (dotted areas) reveal cumulative nuclei (DAPI). Scale bar, 200 µm. **D, E** Representative images and quantification of CC1- and GFAP-positive cells in the lesion (dotted areas). Scale bars, 50 µm (**D**), 100 µm (**E**). **F** mGFP in the SVZ and counter-immunostaining for NG2 (OPCs) and Olig2 (OLs) in the hGFAP-CreERT; mT/mG brain. Scale bar, 50 µm. Error bars represent the mean ± SEM. *P < 0.05, Student’s *t*-test, Dicer-cKO vs control, *n* = 3–4 for all experiments.

To confirm the recombination efficiency in astrocytes driven by hGFAP-CreERT in adulthood, we examined the mGFP expression rate by immunostaining for GFAP in the hGFAP-CreERT; mT/mG mouse at P90, 30 days after induction from P60 (Fig. 6A). Our results showed that mGFP expression was exclusively co-localized with GFAP-positive cells, and approximately 75% of GFAP-positive cells expressed mGFP at P90, indicating astroglia-specific and efficient recombination in the adult CNS (Fig. 6B). To investigate if Dicer deletion in astrocytes could change myelin regeneration in lesions, we induced recombination at the age of 90 days and injected lysolecithin into the corpus callosum 7 days after the recombination (Fig. 6C). Demyelination induced by lysolecithin injection triggers an automatic myelin reparative process in the CNS, that allows for measuring remyelination kinetics in the lesions. The MBP immunostaining results indicated that remyelination in the corpus callosum was greatly decreased 14 days post-injury, suggesting that Dicer deletion in astrocytes also inhibits remyelination after lysolecithin lesions. In support of this finding, the CC1-positive cell density was also significantly lower in the Dicer-cKO lesions than in the wild-type controls (Fig. 6D). Immunostaining for GFAP revealed increased astroglial area of the Dicer-cKO lesion, implying astrogliosis induced by Dicer deletion (Fig. 6E). It was notable that hGFAP-CreERT also induced recombination in the SVZ progenitors, and approximately 10% of SVZ cells expressed mGFP. Since SVZ neural progenitors can migrate to the lesions and differentiate into OPCs, contributing to remyelination, we next examined if any OLs in the lesions were derived from mGFP-positive cells (Fig. 6F). Immunostaining for NG2 or Olig2 did not show any mGFP-positive cells co-expressing NG2 or Olig2 (Fig. 6F), indicating that the SVZ progenitors were unlikely to differentiate into OLs and contribute to remyelination after lysolecithin-induced demyelination in this case. Therefore, our results indicated that Dicer deletion in astrocytes during adulthood caused astrogliosis and inhibited OL differentiation after lysolecithin-induced demyelination.

## Discussion

Astrocytes play an important role in governing CNS development [26, 27]. The functional importance of Dicer in OLs [28], microglia, and neurons has been extensively investigated [29, 30]. Here, we used the hGFAP-CreERT line to induce recombination in the developing and adult CNS. Revealed by the mT/mG reporter line, hGFAP-CreERT can drive an efficient and astrocyte-specific recombination without leaking into other cell types in the postnatal CNS. Previous reports have shown that the conditional Dicer deletion driven by hGFAP-Cre or mGFAP-Cre results in severe functional deficits, such as ataxia and seizures, and premature deaths of the mutant mice [16], while the Dicer deletion in the developing CNS causes memory and motor coordination deficits in adulthood without severe dysfunctions. The differences were probably due to the non-specific Dicer deletion in astrocytes, neurons and OLs by hGFAP-Cre and mGFAP-Cre [19, 31]. It remains largely unknown how Dicer deletion in astrocytes impairs neurofunctions in this case. Given that hypomyelination may disrupt synaptogenesis and cause long-term functional deficits [32] and we demonstrated that Dicer deletion in astrocytes causes hypomyelination in the developing CNS, it is possible that the hypomyelination may also contribute, at least partially, to functional abnormalities in the Dicer-cKO mice. Regarding the functional importance of astrocytes in supporting neurons and regulating neuronal synaptogenesis, it also highly probable that Dicer deletion in astrocytes could directly affect neuronal functions and be involved in the functional deficits in the Dicer-cKO mice [16, 17]. It is notable that Dicer deletion in astrocytes during adulthood seems unlikely to cause instant functional deficits, suggesting that Dicer deletion in adult astrocytes may not impart acute and severe damage to neuronal function.

Evidence has shown that astrocytes and OLs are closely associated during CNS development. Elimination of astrocytes in developing CNS causes OL death and disrupts myelination in the developing CNS, indicating that astrocytes are functionally required for OL survival [15]. Astrocytes can support myelination through both cell-to-cell communication and secretory mechanisms. Previously, we and others have shown that astrocytes can provide nutrients to OPCs by gap junctions, and deletion of Connexin 43 in astrocytes inhibits astrogliosis and promotes remyelination [33–35]. Further, astrocytes can also produce a number of growth factors, like CNTF and PDGF, to support OL proliferation and myelination [36, 37]. Thus, it is also possible that the Dicer deletion in astrocytes might diminish the support for OL differentiation and myelination. Since Dicer deletion can cause astrogliosis and over-activation in the developing and adult CNS, it is plausible that the aberrant astroglial changes may disrupt the supporting functions for OL differentiation and myelination. Together, this study has demonstrated that Dicer in astrocytes is required for OL myelination and neurofunction development.

## References

[1] Sofroniew MV, Vinters HV (2010). Astrocytes: biology and pathology. Acta Neuropathol.

[2] Suzuki A, Stern SA, Bozdagi O, Huntley GW, Walker RH, Magistretti PJ (2011). Astrocyte-neuron lactate transport is required for long-term memory formation. Cell.

[3] Bélanger M, Allaman I, Magistretti PJ (2011). Brain energy metabolism: Focus on astrocyte-neuron metabolic cooperation. Cell Metab.

[4] Falkowska A, Gutowska I, Goschorska M, Nowacki P, Chlubek D, Baranowska-Bosiacka I (2015). Energy metabolism of the brain, including the cooperation between astrocytes and neurons, especially in the context of glycogen metabolism. Int J Mol Sci.

[5] Colangelo AM, Alberghina L, Papa M (2014). Astrogliosis as a therapeutic target for neurodegenerative diseases. Neurosci Lett.

[6] Glass CK, Saijo K, Winner B, Marchetto MC, Gage FH (2010). Mechanisms underlying inflammation in neurodegeneration. Cell.

[7] Sherman DL, Brophy PJ (2005). Mechanisms of axon ensheathment and myelin growth. Nat Rev Neurosci.

[8] Hartline DK, Colman DR (2007). Rapid conduction and the evolution of giant axons and myelinated fibers. Curr Biol.

[9] Miron VE (2017). Microglia-driven regulation of oligodendrocyte lineage cells, myelination, and remyelination. J Leukoc Biol.

[10] Lundgaard I, Osório MJ, Kress BT, Sanggaard S, Nedergaard M (2014). White matter astrocytes in health and disease. Neuroscience.

[11] Orthmann-Murphy JL, Abrams CK, Scherer SS (2008). Gap junctions couple astrocytes and oligodendrocytes. J Mol Neurosci.

[12] Padovani-Claudio DA, Liu L, Ransohoff RM, Miller RH (2006). Alterations in the oligodendrocyte lineage, myelin, and white matter in adult mice lacking the chemokine receptor CXCR2. Glia.

[13] Jiang P, Chen C, Liu XB, Pleasure DE, Liu Y, Deng W (2016). Human iPSC-derived immature astroglia promote oligodendrogenesis by increasing TIMP-1 secretion. Cell Rep.

[14] Meyer-Franke A, Shen S, Barres BA (1999). Astrocytes induce oligodendrocyte processes to align with and adhere to axons. Mol Cell Neurosci.

[15] Tognatta R, Karl MT, Fyffe-Maricich SL, Popratiloff A, Garrison ED, Schenck JK (2020). Astrocytes are required for oligodendrocyte survival and maintenance of myelin compaction and integrity. Front Cell Neurosci.

[16] Howng SY, Huang Y, Ptáček L, Fu YH (2015). Understanding the role of dicer in astrocyte development. PLoS One.

[17] Sun C, Zhu L, Ma R, Ren J, Wang J, Gao S (2019). Astrocytic miR-324-5p is essential for synaptic formation by suppressing the secretion of CCL5 from astrocytes. Cell Death Dis.

[18] Felts PA, Woolston AM, Fernando HB, Asquith S, Gregson NA, Mizzi OJ (2005). Inflammation and primary demyelination induced by the intraspinal injection of lipopolysaccharide. Brain.

[19] Hong P, Jiang M, Li H (2014). Functional requirement of dicer1 and miR-17-5p in reactive astrocyte proliferation after spinal cord injury in the mouse. Glia.

[20] Hirrlinger PG, Scheller A, Braun C, Hirrlinger J, Kirchhoff F (2006). Temporal control of gene recombination in astrocytes by transgenic expression of the tamoxifen-inducible DNA recombinase variant CreERT2. Glia.

[21] Wang F, Ren SY, Chen JF, Liu K, Li RX, Li ZF (2020). Myelin degeneration and diminished myelin renewal contribute to age-related deficits in memory. Nat Neurosci.

[22] Vorhees CV, Williams MT (2006). Morris water maze: Procedures for assessing spatial and related forms of learning and memory. Nat Protoc.

[23] Brooks SP, Dunnett SB (2009). Tests to assess motor phenotype in mice: A user's guide. Nat Rev Neurosci.

[24] Mei F, Mayoral SR, Nobuta H, Wang F, Desponts C, Lorrain DS (2016). Identification of the kappa-opioid receptor as a therapeutic target for oligodendrocyte remyelination. J Neurosci.

[25] Mei F, Wang H, Liu S, Niu J, Wang L, He Y (2013). Stage-specific deletion of Olig2 conveys opposing functions on differentiation and maturation of oligodendrocytes. J Neurosci.

[26] Molofsky AV, Krencik R, Ullian EM, Tsai HH, Deneen B, Richardson WD (2012). Astrocytes and disease: A neurodevelopmental perspective. Genes Dev.

[27] Liu JH, Li ZL, Liu YS, Chu HD, Hu NY, Wu DY (2020). Astrocytic GABAB receptors in mouse *Hippocampus* control responses to behavioral challenges through astrocytic BDNF. Neurosci Bull.

[28] Li T, Wang J, Wang H, Yang Y, Wang S, Huang N (2018). The deletion of dicer in mature myelinating glial cells causes progressive axonal degeneration but not overt demyelination in adult mice. Glia.

[29] Varol D, Mildner A, Blank T, Shemer A, Barashi N, Yona S (2017). Dicer deficiency differentially impacts microglia of the developing and adult brain. Immunity.

[30] Chmielarz P, Konovalova J, Najam SS, Alter H, Piepponen TP, Erfle H (2017). Dicer and microRNAs protect adult dopamine neurons. Cell Death Dis.

[31] Chen X, Wang F, Gan J, Zhang Z, Liang X, Li T (2020). Myelin deficits caused by Olig2 deficiency lead to cognitive dysfunction and increase vulnerability to social withdrawal in adult mice. Neurosci Bull.

[32] Wang F, Yang YJ, Yang N, Chen XJ, Huang NX, Zhang J (2018). Enhancing oligodendrocyte myelination rescues synaptic loss and improves functional recovery after chronic hypoxia. Neuron.

[33] Li T, Niu J, Yu G, Ezan P, Yi C, Wang X (2020). Connexin 43 deletion in astrocytes promotes CNS remyelination by modulating local inflammation. Glia.

[34] Rash JE, Yasumura T, Dudek FE, Nagy JI (2001). Cell-specific expression of connexins and evidence of restricted gap junctional coupling between glial cells and between neurons. J Neurosci.

[35] Niu JQ, Li T, Yi CJ, Huang NX, Koulakoff A, Weng CH (2016). Connexin-based channels contribute to metabolic pathways in the oligodendroglial lineage. J Cell Sci.

[36] Stankoff B, Aigrot MS, Noël F, Wattilliaux A, Zalc B, Lubetzki C (2002). Ciliary neurotrophic factor (CNTF) enhances myelin formation: A novel role for CNTF and CNTF-related molecules. J Neurosci.

[37] Modi KK, Sendtner M, Pahan K (2013). Up-regulation of ciliary neurotrophic factor in astrocytes by aspirin: Implications for remyelination in multiple sclerosis. J Biol Chem.

